# The Role of Estrogen and ER Stress in Glycemic Regulation in the Sexually Dimorphic TALLYHO/JngJ Mouse Model of Diabetes

**DOI:** 10.1210/jendso/bvaf048

**Published:** 2025-03-15

**Authors:** Monica De Paoli, Zinal Patel, Susanna Fang, Geoff H Werstuck

**Affiliations:** Thrombosis and Atherosclerosis Research Institute, Hamilton, ON, Canada L8L 2X2; Department of Biochemistry and Biomedical Sciences, McMaster University, Hamilton, ON, Canada L8S 4K1; Department of Medicine, McMaster University, Hamilton, ON, Canada L8S 4K1; Department of Medicine, McMaster University, Hamilton, ON, Canada L8S 4K1; Thrombosis and Atherosclerosis Research Institute, Hamilton, ON, Canada L8L 2X2; Department of Biochemistry and Biomedical Sciences, McMaster University, Hamilton, ON, Canada L8S 4K1; Department of Medicine, McMaster University, Hamilton, ON, Canada L8S 4K1

**Keywords:** hyperglycemia, endoplasmic reticulum stress, estrogen, unfolded protein response

## Abstract

The global incidence of diabetes mellitus is increasing, causing a heavy burden on health care management and costs. Sex differences in the incidence and prevalence of diabetes mellitus do exist, with premenopausal women being protected from developing this disease, compared to men or postmenopausal women. The mechanisms underlying these differences are not yet known and experimental animal models can significantly advance our understanding of these processes. In this study we characterized a mouse model of polygenic type 2 diabetes, the TALLYHO/JngJ mouse, which shows sexual dimorphism in blood glucose regulation. Male TALLYHO/JngJ mice develop chronic hyperglycemia by 5 weeks of age, while females remain normoglycemic. We analyzed the role of endoplasmic reticulum (ER) stress and the activation of the unfolded protein response (UPR) in the development of hyperglycemia in this mouse model. Additionally, we evaluated the effect of estrogen depletion in female TALLYHO/JngJ mice through ovariectomies. Ovariectomized female mice and males become chronically hyperglycemic (fasting blood glucose threshold >15 mM) and show significantly increased expression of GRP78/GRP94, markers of the adaptive unfolded protein response (UPR). GADD153/CHOP, a marker of the apoptotic UPR, is significantly increased in ovariectomized female mice. Treatment with a chemical chaperone 4-PBA, an ER stress alleviator, improves but does not normalize blood glucose levels in male and ovariectomized female TALLYHO/JngJ mice. Together, these findings support a protective role for estrogen and identify the UPR as a pathway through which estrogen may maintain pancreatic beta cell health.

Physiological levels of blood glucose are primarily regulated by the peptide hormones insulin and glucagon, produced by pancreatic beta and alpha cells respectively. When proper homeostasis cannot be maintained there is an increased risk of developing type 2 diabetes mellitus, a metabolic disorder characterized by chronic hyperglycemia. The incidence of type 2 diabetes has been steadily growing in recent years, and it is expected to soar over the next 20 years due to a significant increase of predisposing risk factors, such as overweight and obesity, and a sedentary lifestyle among the general population [1, 2]. Individuals with type 2 diabetes have an increased risk of developing cardiovascular complications, such as myocardial infarction and stroke, highlighting the significant burden posed by this disease and its complications in terms of patient management and health care costs [1-4].

The endoplasmic reticulum (ER) is a cellular organelle responsible for the synthesis, proper folding, and maturation of proteins that are destined for secretion or transport to the plasma membrane. Pancreatic beta cells have a well-structured ER to support their ability to synthesize and secrete appropriate amounts of insulin into the circulation [5]. Conditions of chronic hyperglycemia induce pancreatic beta cells to synthesize large amounts of insulin in a short period of time. This can lead to an overwhelmed ER, where the protein-folding capability of the cell is exceeded [6-11] This imbalance is known as ER stress. Cells respond to ER stress by activating the unfolded protein response (UPR) in order to restore homeostasis [12]. The UPR can be activated through 3 ER-resident transmembrane proteins: protein kinase-like ER kinase (PERK), activating transcription factor 6 (ATF6), and inositol-requiring enzyme 1α (IRE1α) [13, 14]. These activators initially function to restore cell homeostasis by increasing the protein folding capacity of the ER. This is accomplished through the upregulation of expression of protein chaperones/foldases, such as glucose regulated protein (GRP)78, GRP 94, and protein disulfide isomerase (PDI), and by enhancing ER-associated degradation (ERAD) of terminally misfolded proteins. These processes are collectively known as the adaptive UPR [13]. However, when ER stress is protracted and ER homeostasis cannot be achieved, such as in conditions of chronic hyperglycemia, apoptotic signals are initiated, and the cell undergoes programmed cell death. This is referred to as the apoptotic UPR [12, 13, 15, 16]. Although several studies have investigated the link between ER stress and the development of hyperglycemia and diabetes, the specific mechanisms by which this occurs are still unknown.

Rodent models of chronic hyperglycemia can be useful to investigate the mechanisms underlying these metabolic disorders [17, 18]. In this experimental study we characterize the TALLYHO/JngJ (TH) mouse model, which is a polygenic model of type 2 diabetes and obesity showing sexual dimorphism in glucose regulation [19, 20]. These mice have been selected for hyperglycemia over many generations from a colony of outbred mice in which some male mice spontaneously developed hyperglycemia, glycosuria, and polyuria [19]. Male TH mice develop chronic hyperglycemia, whereas female TH mice remain normoglycemic throughout their lifespan [19, 20]. Thus, the sexual dimorphism in this rodent strain is an extreme model of what is observed in humans with respect to development and progression of type 2 diabetes, with premenopausal women appearing to be less susceptible to developing diabetes, compared to men or postmenopausal women [21-23].

The aim of this study is to characterize this mouse model, and to better understand the role of ER stress in the development of hyperglycemia, evaluating potential sex differences in the modulation and/or activation of the UPR.

## Materials and Methods

### Mouse Model

In this study we used the TALLYHO/JngJ (TH) mouse model characterized by moderate obesity and spontaneous chronic hyperglycemia in males, but not females [19]. Male and female TH mice were purchased from The Jackson Laboratory (JAX stock #005314). Characterization and experimental procedures started from the F7NE4F7 + 2 breeding generation.

Experimental mice received a standard diet (2018 Teklad Global 18% Protein Rodent Diet, Harlan Teklad, Madison, WI, USA) ad libitum, and free access to water. As the hyperglycemic phenotype is not fully penetrant, male mice were checked for hyperglycemia at 8 weeks of age. Male mice that did not meet the hyperglycemic threshold (fasting blood glucose levels >15 mM) were eliminated from the study. All animal procedures were pre-approved by the McMaster University Animal Research Ethics Board.

### Ovariectomy

Ovariectomy was performed on subsets of female TH mice (n = 5-10 per experimental group) at 4 weeks of age using previously described methods [24, 25]. Briefly, a 3 × 3 cm incision area surrounding the iliac crest was shaved and cleaned, and a horizontal incision through the skin in the midline area was performed. Once the ovary was identified, an incision was made through the muscle layer to reach the abdominal cavity. The fat surrounding the ovary was removed and the ovary was exposed, a double ligation was performed in the uterine horn and vessels, and the ovary was removed. The remaining tissue was put back in the abdominal cavity, and the incision was sutured. The same procedure was used to remove the contralateral ovary. The skin wound was closed using a wound clipper. Sham-operated animals (n = 5-10 per experimental group) underwent a similar procedure; however, ovaries were not removed.

### Treatment With 4-Phenylbutyric Acid

Subsets of female mice were either sham-operated or ovariectomized at 4 weeks of age. At 5 weeks of age, male, sham female, and ovariectomized female TH mice were switched to drinking water containing 4-phenylbutyric acid (4-PBA) for 15 weeks (n = 5-10 per experimental group). 4-PBA is a chemical chaperone that prevents misfolded protein aggregation, thereby alleviating ER stress [26]. Control male, sham female, and ovariectomized female TH mice (n = 5-10 per experimental group) received regular drinking water. The 4-PBA was administered at a concentration of 3.8 g/L of drinking water, corresponding to a dosage of 1 g 4-PBA/kg body weight/day taking into account water intake per mouse, as previously described [27].

Fasting blood glucose levels were measured at 5, 10, 15 and 20 weeks of age. The experimental endpoint was set at 20 weeks of age, where organs and tissues were harvested and analyzed.

### Harvesting

Experimental mice were anesthetized with isoflurane and euthanized by cervical dislocation. Blood was collected and the vasculature was rinsed with phosphate buffered saline. Pancreata were collected and fixed in 10% neutral-buffered formalin and stored at room temperature.

### Glucose Measurements, Glucose Tolerance Test, Lipid Analysis and Serum Insulin Quantification

Fasting blood glucose levels were measured using a glucometer (One Touch Verio Flex, LifeScan, Burnaby, BC, Canada) after mice were fasted as follows: 1-hour fasting for 3- and 4-week-old mice; 2-hours fasting for 5- to 6-week-old mice; 4- to 6-hours fasting for >6-week-old mice.

Subsets of experimental mice (n = 4-6 per experimental group) were fasted at experimental endpoint (5 weeks of age or 20 weeks of age) for 4 to 6 hours. Fasting blood glucose levels (time 0) were determined, and a solution of glucose (2 g/kg body weight of a 200 mg/mL solution) was administered via intraperitoneal (IP) injection. Blood glucose levels were measured after 30, 60 and 120 minutes using a glucometer (One Touch Verio Flex, LifeScan, Burnaby, BC, Canada). Because the glucometer has a maximum detection limit of 35 mM, blood samples exceeding 35 mM were determined using a colorimetric glucose assay (Infinity Glucose Hexokinase Reagent, Thermo Fisher, Middletown, VA, USA). Fasting plasma lipid levels were quantified using the colorimetric diagnostic kits for total cholesterol and triglyceride determination (Infinity Triglyceride, Infinity Cholesterol, Thermo Scientific, Middletown, VA). Fasting serum insulin was determined through an enzyme-linked immunosorbent assay (Ultra Sensitive Mouse Insulin ELISA Kit, 90080, Chrystal Chem, Elk Grove Village, IL 60007 USA, RRID:AB_3662574).

### Pancreata Analysis

Pancreata from male, sham-operated female, and ovariectomized female TH mice were harvested at 20 weeks of age. Pancreata were embedded in paraffin and 6-µm thick sections were cut using a microtome. The entire pancreas was analyzed at 180-µm intervals for a comprehensive overview of the islets within the organ. A total of 6 sections per mouse were used to perform immunohistochemical and immunofluorescent analysis, and a total of n = 12 to n = 20 islets per mouse were selected for the intensity of fluorescence analysis. A total of n = 5 to n = 8 mice per experimental group were analyzed.

Immunofluorescence intensity was visualized, and images were captured using a Leica STELLARIS 5 confocal microscope. Analysis was performed using ImageJ software (NIH, Bethesda, MD, USA; http://imagej.nih.gov/ij). The intensity of fluorescence staining for each mouse was calculated as follows:

Intensity of fluorescence = (average intensity of fluorescence of the pancreatic islets per mouse adjusted for islet area) − (average intensity of fluorescence of the pancreatic islets of the negative control adjusted for islet area).

All immunofluorescent staining experiments were counterstained with DAPI (Invitrogen, Carlsbad, CA, USA) at a dilution of 1:5000. Separate sections were stained with pre-immune IgG, instead of the primary antibody, to control for nonspecific staining. Immunohistochemical analysis was captured using a Leitz LABORLUX S microscope connected to a DP71 Olympus camera. Quantification of the UPR marker GADD153/CHOP was performed by counting the brown-stained nuclei vs the total number of nuclei in the islet (n = 12-20 islets per mouse; n = 4-8 mice per experimental group).

Immunohistochemical analysis was performed for the quantification of alpha and beta cell mass and for the assessment of GADD153/CHOP. Immunofluorescent staining was performed to determine insulin and glucagon content and the quantification of the UPR markers GRP78/GRP94, PDI, and ATF4. The following dilutions and antibodies were used: 1:100 dilution for monoclonal rat IgG insulin antibody (MAB1417, R&D Systems, Minneapolis, MN, USA, RRID:AB_3659005) and polyclonal mouse glucagon antibody (CL8867AP-S, Burlington, ON, Canada, RRID: AB_3675724); 1:50 dilution for GADD153/CHOP (monoclonal mouse GADD153 (B3) sc-7531, Santa Cruz Biotechnology, Dallas, TX, USA, RRID:AB_634535); 1:250 dilution for GRP78/GRP94 (monoclonal mouse KDEL antibody, ADI-SPA-827-J, Enzo/Cedarlane, Burlington, ON, Canada, RRID: AB_3675726); 1:200 dilution for PDI (monoclonal mouse antibody, ADI-SPA-891-F, Enzo/Cedarlane, Burlington, ON, Canada, RRID: AB_3675727) and ATF4 (polyclonal rabbit antibody, 10835-1-AP, Thermo Fischer, Mississauga, ON, Canada, RRID:AB_2058600). A 1:200 dilution was used for the secondary antibodies: goat anti-rat biotinylated IgG (BA 9400, Vector, Burlington, ON, Canada, RRID:AB_3107017), goat anti-rabbit biotinylated IgG (BA 1000-1.5, Vector, Burlington, ON, Canada, RRID:AB_2313606), goat anti-mouse biotinylated IgM (BA 2020, Vector, Burlington, ON, Canada, RRID:AB_2336183), Alexa Fluor 488 goat anti-rat IgG (A11006, Thermo Scientific, Middletown, VA, USA, RRID:AB_2534074), Alexa Fluor 568 goat anti-mouse IgG (A11004, Thermo Scientific, Middletown, VA, USA, RRID:AB_2534072), Alexa Fluor 488 goat anti-mouse IgG (A11001, Thermo Scientific, Middletown, VA, USA, RRID:AB_2534069), Alexa Fluor 488 goat anti-rabbit IgG (A11008, Thermo Scientific, Middletown, VA, USA, RRID: AB_3675729). Separate sections were stained with pre-immune IgG instead of the primary antibody, to control for nonspecific staining. Immunohistochemistry used the developer reagent DAB+ Substrate Chromogen System (DAKO, K3468, Agilent Technologies, Santa Clara, CA, USA) as directed by the manufacturer.

### Quantification of Alpha and Beta Cell Mass

Alpha cell mass (ACM) was calculated using the following formula [28]:


$$\begin{array}{rl} ACM\;= & (average\;of\;total\;glucagon\text{-}positive\;stained\;area/\\ & total\;section\;area)\times pancreatic\;mass\;(mg) \end{array}$$

Beta cell mass (BCM) was calculated using the following formula [28]:


$$\begin{array}{rl} BCM\;= & (average\;of\;insulin\text{-}positive\;stained\;area:\;total\\ & area\;of\;section)\times pancreatic\;mass\;(mg) \end{array}$$

Glucagon and insulin content of pancreatic islets were determined by assessing immunofluorescence intensity using the previously described antibody against insulin (1:100 dilution) and polyclonal mouse glucagon antibody (CL8867AP-S, Burlington, ON, Canada) (1:200 dilution), and counterstained with DAPI (Invitrogen, Carlsbad, CA, USA) at a dilution of 1:5000. Immunostaining was detected using secondary antibodies Alexa Fluor 488 goat anti-rat IgG (A11006, Thermo Scientific, Middletown, VA, USA), Alexa Fluor 568 goat anti-mouse IgG (A11004, Thermo Scientific, Middletown, VA, USA) each at 1:200 dilution. Separate sections were stained with pre-immune IgG instead of the primary antibody, to control for nonspecific staining. Six pancreatic sections per mouse were analyzed and a total of n = 15 to 33 islets per male mouse and n = 17 to 37 islets per female mouse were selected. Images were captured using a Leica DMi8 confocal microscope at 20× magnification, and the intensity of fluorescence analysis was performed with ImageJ software. The intensity of fluorescence staining for each mouse was calculated as follows: (average intensity of fluorescence of the total number of islets per mouse) − (average intensity of fluorescence of the total number of islets in the negative control). No adjustments to intensity or contrast were made to any of the images analyzed.

### Statistical Analysis

Statistical analyses for multiple groups were performed using a *t* test, one-way or two-way ANOVA, as appropriate. Data are presented as mean ± standard error of the mean (SEM). Analyses were performed using ImageJ. A significance value of *P* < .05 was considered statistically significant.

## Results

### Fasting Glucose Levels in TH Mice

**Figure 1.**
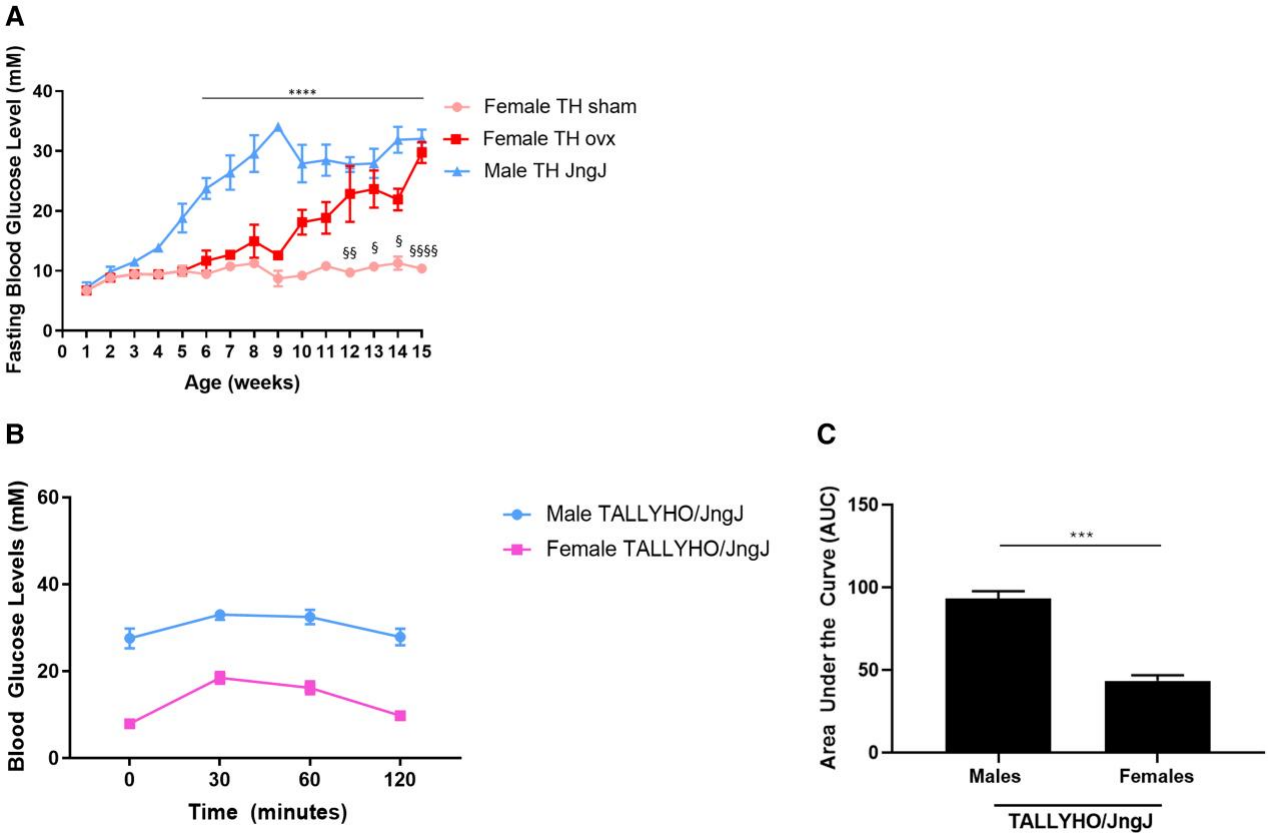
(A) Fasting blood glucose levels in female sham-operated and ovariectomized (ovx) and age-matched male TALLYHO/JngJ (TH). ****P < .0001 male TH vs age-matched female TH sham. § *P* < .05 female TH sham vs age-matched female TH ovx, §§ *P* < .01 female TH sham vs age-matched female TH ovx, §§§§ *P* < .0001 female TH sham vs age-matched female TH ovx. n = 4 to 10 per experimental group. Bars represent standard error of the mean (SEM). (B, C) Glucose tolerance test in 5-week-old female and male TH. ***P < .001; n = 4 per experimental group. Bars represent SEM. Average glucose levels (mmol/L) female TALLYHO/JngJ: 7.9 (*0 minutes*); 18.47 (*30 minutes*); 16.20 (*60 minutes*); 9.75 (*120 minutes*). Average glucose levels (mmol/L) male TALLYHO/JngJ: 27.55 (*0 minutes*); 33 (*30 minutes*); 32.47 (*60 minutes*); 27.87 (*120 minutes*).

In this study we observed that male TH mice develop chronic hyperglycemia by 5 weeks of age, whereas female mice maintain the normoglycemic phenotype throughout their lifespan (Fig. 1). Glucose tolerance tests were performed in 5-week-old male and female TH mice (Fig. 1B and 1C). Female TH mice tolerate a glucose challenge significantly better than age-matched male TH mice. To investigate the potential effects of estrogen depletion in glucose regulation, a subset of female TH mice underwent ovariectomy. When ovariectomized, female TH mice developed chronic hyperglycemia by 12 weeks of age.

To investigate the potential role of ER stress in the development of hyperglycemia in this mouse model, 5-week-old male, female sham-operated, and female ovariectomized TH mice received either regular drinking water or water containing 4-PBA.

### Metabolic Parameters in TH Mice

There were no significant differences in body weight or pancreas weight between any of the experimental groups of female mice (Table 1). Liver weight was significantly increased in female sham-operated mice that received treatment with 4-PBA, compared to those receiving regular water. No significant differences in liver weight were observed in the ovariectomized groups. Adipose tissue weight was significantly increased in the female sham-operated mice receiving regular water compared to the ovariectomized counterparts. Ovariectomized mice presented with increased plasma cholesterol and triglyceride levels compared to sham-operated counterparts.

**Table 1.** Metabolic parameters of female TALLYHO/JngJ either treated or not with 4-PBA

Mouse genotype/intervention	Body weight at 20 weeks (g)	Pancreas weight (g)	Liver weight (g)	Adipose tissue weight (g)	Plasma cholesterol (mM)	Plasma triglycerides (mM)	Serum insulin (ng/mL)
TALLYHO/JngJ sham	43.80 ± 3.10	0.22 ± 0.01	1.51 ± 0.15	3.59 ± 0.82	3.73 ± 0.99*^a^*	1.02 ± 0.23*^b^*	0.90 ± 0.16*^c^*
TALLYHO/JngJ sham + 4-PBA	44.14 ± 1.81	0.24 ± 0.07	2.37 ± 0.36*^d^*	4.98 ± 0.20*^e^*	3.29 ± 1.39*^f^*	1.07 ± 0.12*^g^*	0.63 ± 0.16
TALLYHO/JngJ ovx	39.94 ± 1.42	0.21 ± 0.02	2.03 ± 0.06	2.36 ± 0.34	5.40 ± 0.52	1.97 ± 0.26	0.35 ± 0.15
TALLYHO/JngJ ovx + 4-PBA	43.99 ± 1.25	0.21 ± 0.01	2.14 ± 0.06	3.26 ± 0.16	6.88 ± 0.60*^h^*	1.21 ± 0.22	0.47 ± 0.15

Each average value is presented with the standard error of the mean. Abbreviation: 4-PBA, 4-phenylbutyric acid.

^a^ *P* < 0.001 TALLYHO/JngJ sham vs TALLYHO/JngJ ovx + 4-PBA.

^b^ *P* < .0001 TALLYHO/JngJ sham vs TALLYHO/JngJ ovx.

^c^ *P* < .05 TALLYHO/JngJ sham vs TALLYHO/JngJ ovx n = 4-9/group.

^d^ *P* < .05 TALLYHO/JngJ sham vs TALLYHO/JngJ sham + 4-PBA.

^e^ *P* < .01 TALLYHO/JngJ sham + 4-PBA vs TALLYHO/JngJ ovx.

^f^ *P* < .0001 TALLYHO/JngJ sham + 4-PBA vs TALLYHO/JngJ ovx + 4-PBA.

^g^ *P* < .0001 TALLYHO/JngJ sham +4-PBA vs TALLYHO/JngJ ovx + 4-PBA.

^h^ *P* < .05 TALLYHO/JngJ sham + 4-PBA vs TALLYHO/JngJ ovx + 4-PBA.

Male TH mice receiving regular water had significantly increased pancreas weight and adipose tissue weight compared to those receiving treatment with 4-PBA (Table 2). Conversely, males receiving treatment with 4-PBA had increased liver weights compared to respective controls. No significant differences were observed for body weight, plasma cholesterol, or plasma triglyceride levels.

**Table 2.** Metabolic parameters of male TALLYHO/JngJ either treated or not with 4-PBA.

Mouse genotype/intervention	Body weight at 20 weeks (g)	Pancreas weight (g)	Liver weight (g)	Adipose tissue weight (g)	Plasma cholesterol (mM)	Plasma triglycerides (mM)	Serum insulin (ng/mL)
TALLYHO/JngJ	29.26 ± 0.44	0.17 ± 0.01*^a^*	0.97 ± 0.29*^b^*	0.35 ± 0.07*^a^*	3.47 ± 1.29	2.91 ± 1.09	0.29 ± 0.1
TALLYHO/JngJ + 4-PBA	32.11 ± 1.59	0.14 ± 0.01	3.58 ± 0.18	0.17 ± 0.02	5.15 ± 1.45	2.31 ± 0.83	0.19 ± 0.1

Each average value is presented with the standard error of the mean. n = 5 to 9/group.

^a^ *P* < .05.

^b^ *P* < .0001.

### Fasting Glucose Levels and Glucose Tolerance in TH Mice Treated With 4-PBA

**Figure 2.**
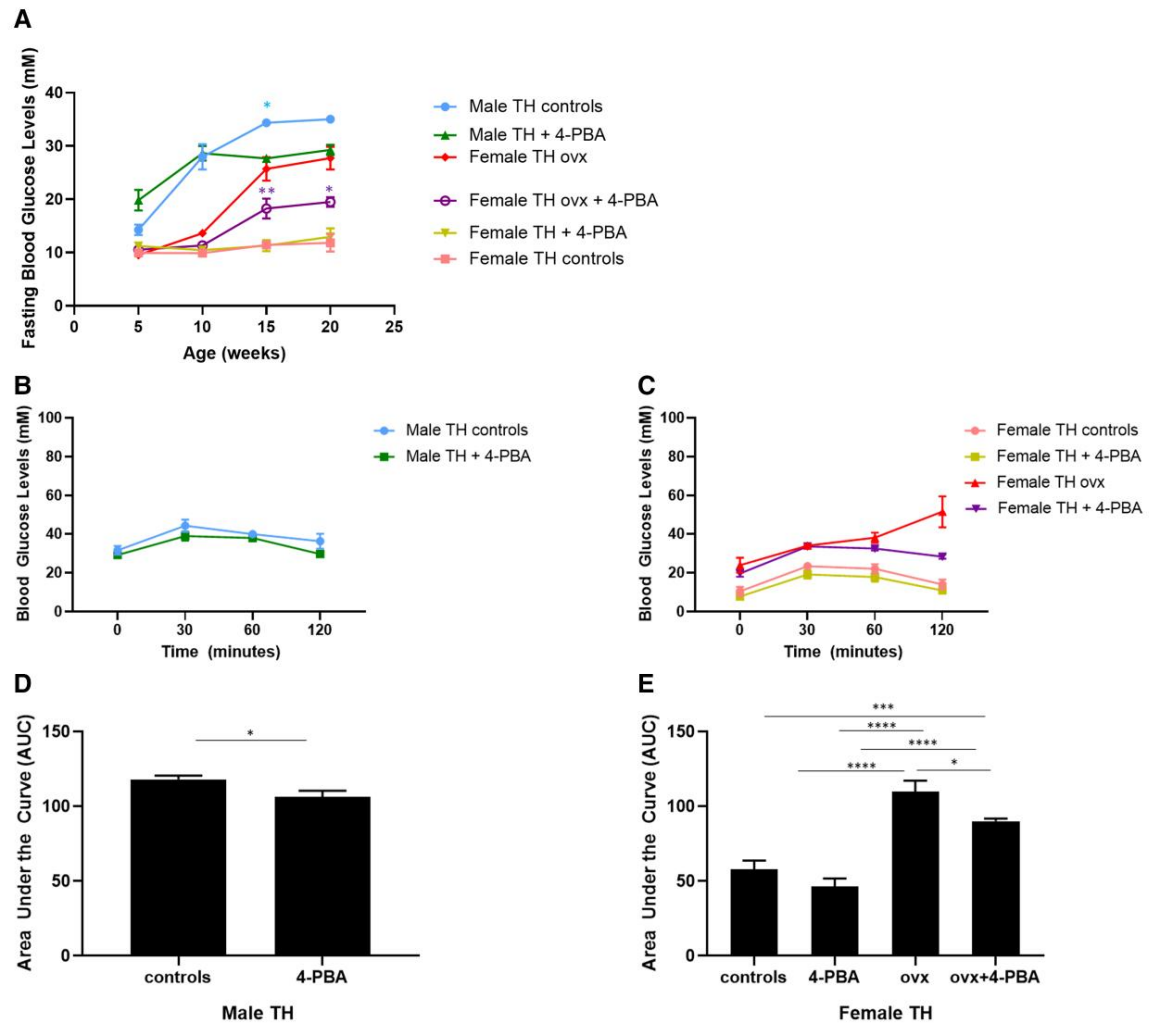
(A) Fasting blood glucose levels in female sham-operated, ovariectomized (ovx) and age-matched male TALLYHO/JngJ either treated or not with the chemical chaperone 4-PBA. *P < .05 male TH control vs age-matched male TH treated with 4-PBA. *P < .05 female TH ovx vs age-matched female TH ovx treated with 4-PBA, **P < .01 female TH ovx vs age-matched female TH ovx treated with 4-PBA, n = 4 to 10 per group. Bars represent standard error of the mean (SEM). Glucose tolerance test (GTT) in 20-week-old male (A, C) and age-matched female sham-operated, ovariectomized (ovx) (B, D) TALLYHO/JngJ either treated or not with the chemical chaperone 4-PBA. *P < .05, ***P < .001, ****P < .0001. n = 4 per group. Bars represent SEM.

Fasting glucose levels in male mice treated with 4-PBA were transiently improved at 15 weeks of age relative to males receiving regular water (Fig. 2). This effect was not significant in 20-week-old male mice. Supplementation with 4-PBA had no effect on normoglycemic female mice. Ovariectomized female TH mice treated with 4-PBA presented with significantly reduced fasting blood glucose levels compared to ovariectomized female controls; however, these mice had significantly higher blood glucose levels compared to intact females. Supplementation with 4-PBA significantly improved glucose tolerance in both male and ovariectomized female TH mice compared to controls (Fig. 2).

### The Effect of 4-PBA on Insulin Production

**Figure 3.**
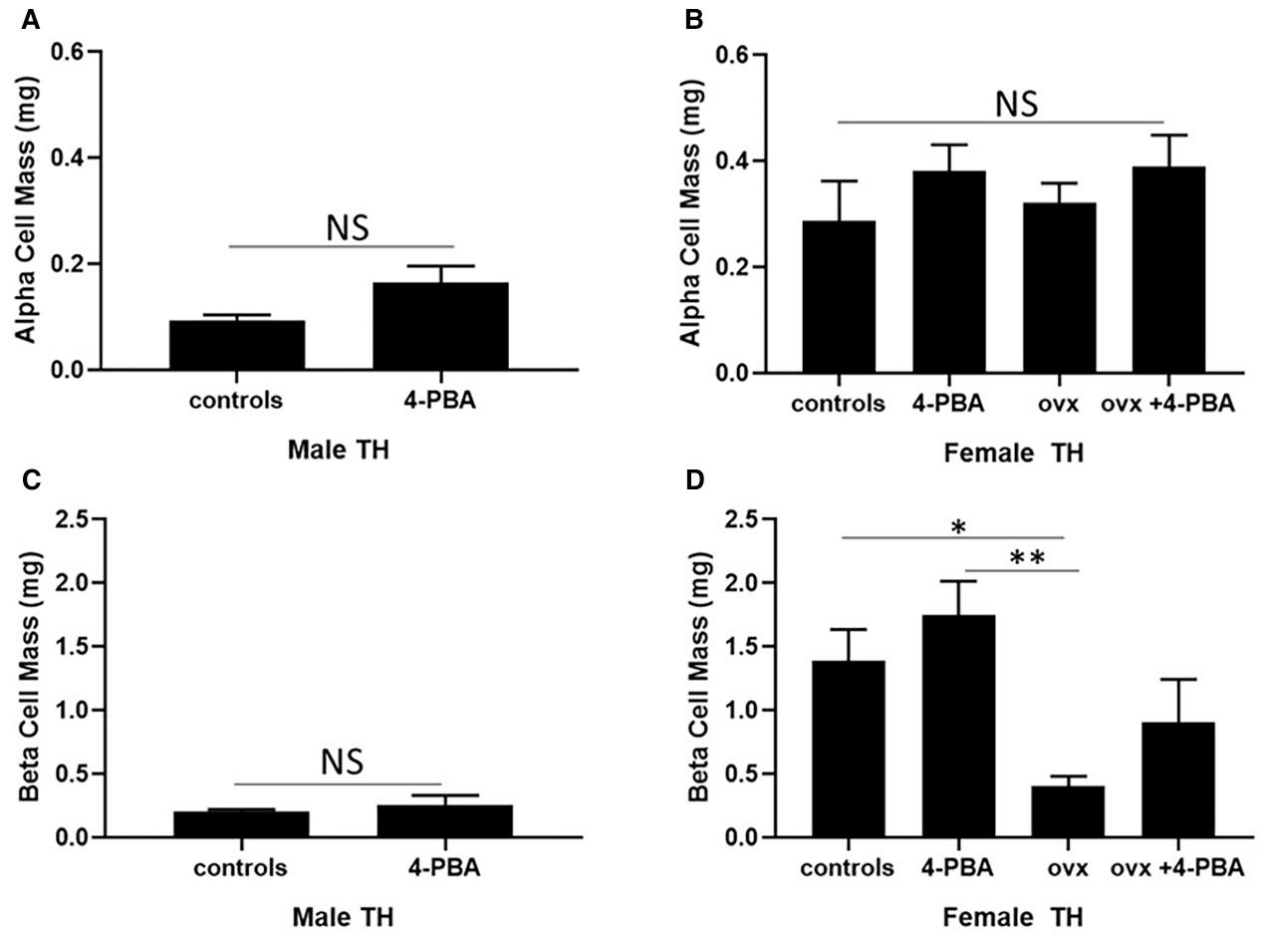
Alpha and beta cell mass in 20-week-old (A, C) male and age-matched (B, D) female sham-operated, ovariectomized (ovx) TH mice either treated or not with the chemical chaperone 4-PBA. n = 5 per group. *P < .05, **P < .01. Bars represent standard error of the mean (SEM). Abbreviation: NS, nonsignificant.

Alpha and beta cell mass were assessed in all experimental groups at 20 weeks of age. Supplementation with 4-PBA did not significantly affect alpha or beta cell mass in male TH mice (Fig. 3A). Consistent with these findings, there was no significant difference in insulin and glucagon content or plasma insulin levels in treated or control male TH mice (Supplemental material [29]).

Alpha cell mass was not significantly affected by ovariectomy or 4-PBA supplementation in female TH mice (Fig. 3B). No significant differences were observed in glucagon content among all female experimental groups (Supplemental material [29]). Beta cell mass was significantly reduced in ovariectomized TH mice, compared to sham-operated female TH mice. Supplementation with 4-PBA did not significantly alter beta cell mass in control or ovariectomized female TH mice. Female ovariectomized mice showed a significant reduction in insulin content, compared to female TH controls (Supplemental material [29]). Plasma insulin levels were significantly reduced in the ovariectomized TH group compared to the sham-operated females (Table 3).

**Table 3.** Fasting serum insulin levels in 20-week-old female TALLYHO/JngJ mice either treated or not with 4-PBA

Mouse genotype/intervention	Serum insulin (ng/mL)
TALLYHO/JngJ sham	0.90 ± 0.16*^a^*
TALLYHO/JngJ sham + 4-PBA	0.63 ± 0.16
TALLYHO/JngJ ovx	0.35 ± 0.15
TALLYHO/JngJ ovx + 4-PBA	0.47 ± 0.15

Each value is presented with the standard error of the mean. n = 5 per experimental group. Abbreviation: 4-PBA, 4-phenylbutyric acid.

^a^ *P* < .05 TALLYHO/JngJ sham vs TALLYHO/JngJ ovx.

### Evaluation of the UPR in TH Mice Treated With 4-PBA

**Figure 4.**
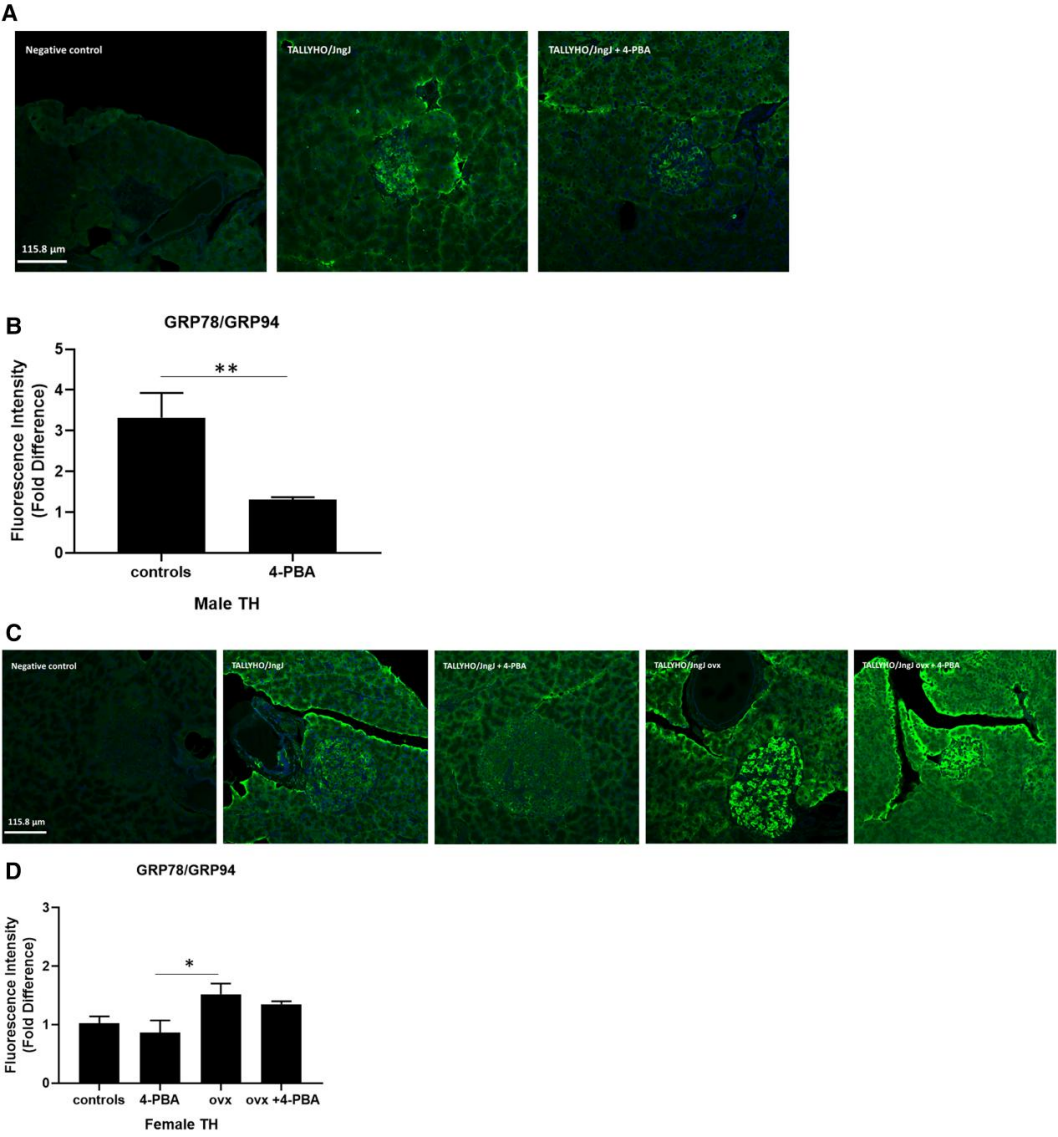
Expression of adaptive UPR markers GRP78/GRP94 in pancreatic islet sections of (A, B) male and (C, D) female sham, female ovariectomized (ovx) TALLYHO/JngJ mice either treated or not with 4-PBA. n = 4 to 5 per group. *P < .05, **P < .01. Bars represent standard error of the mean (SEM).

In order to assess the effects of estrogen and 4-PBA on ER stress in beta cells, markers of the adaptive and apoptotic UPR were quantified in the pancreata of 20-week-old TH mice. GRP78 is an ER-resident chaperone involved in aiding proper protein folding and a key component of the adaptive UPR [30]. GRP78 expression was significantly increased in female ovariectomized mice compared to sham-operated females treated with 4-PBA (Fig. 4). Male mice receiving regular drinking water also had significantly higher levels of GRP78 compared to males treated with 4-PBA.

Protein disulfide isomerase (PDI) also resides in the ER, and mediates the formation of disulfide bonds, which are a key step for proper folding and maturation of insulin [31]. There were no significant differences in PDI expression across female TH experimental groups; however, male mice receiving regular water significantly expressed higher levels of PDI, compared to male TH receiving 4-PBA treatment (Supplemental material [29]).

**Figure 5.**
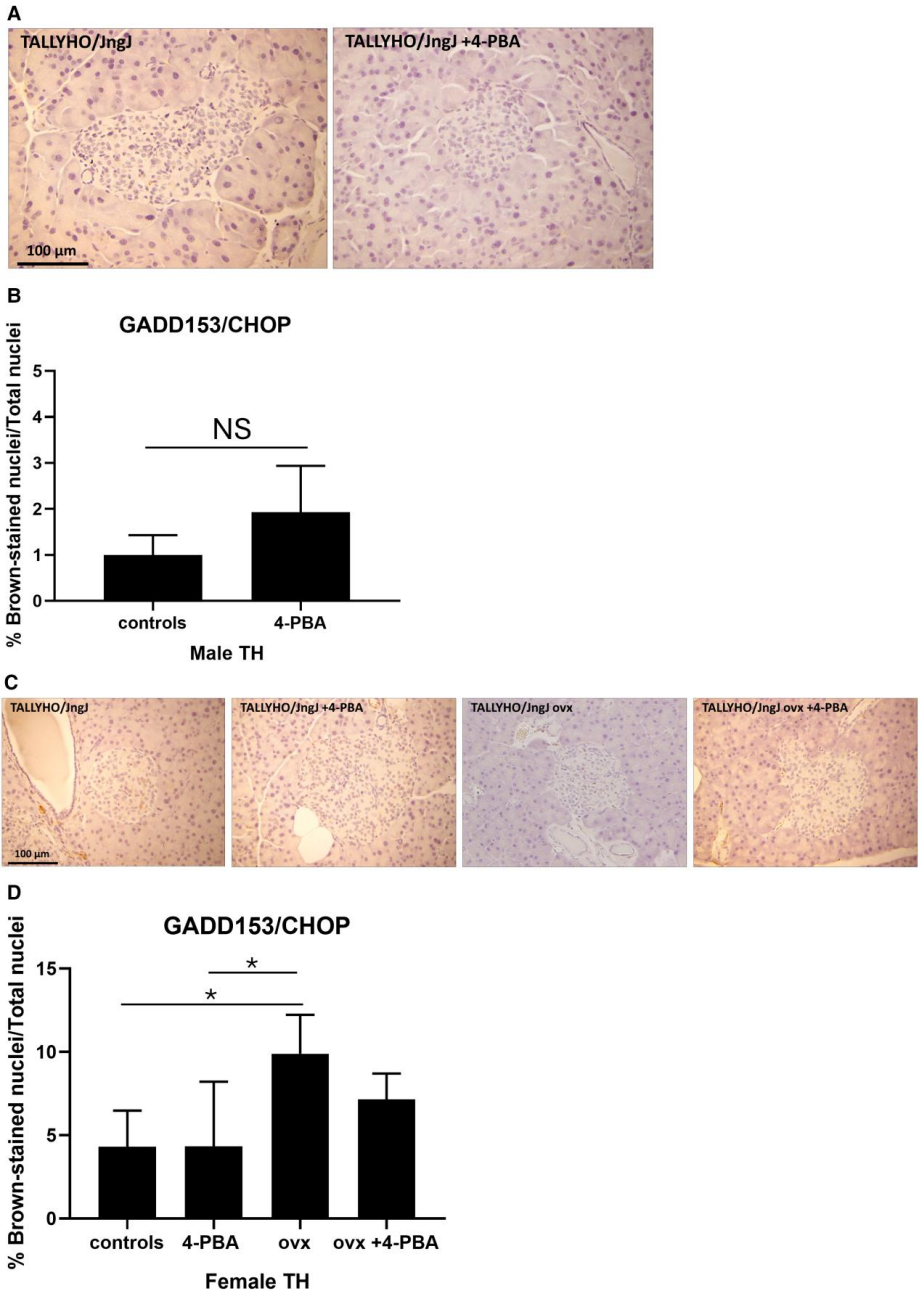
Expression of apoptotic UPR marker GADD153/CHOP in pancreatic islet sections of (A, B) male, (C, D) female sham or ovariectomized TALLYHO/JngJ mice either treated or not with 4-PBA. n = 4 to 5 per group. *P < .05. Bars represent standard error of the mean (SEM). Abbreviation: NS, nonsignificant.

Activating transcription factor 4 (ATF4) is an apoptotic UPR factor that induces the transcription of GADD153/CHOP, which in turn signals the cell to undergo apoptosis. In this study there were no significant differences observed in the expression of ATF4 in either male or female experimental groups (Supplemental material [29]). The expression of GADD153/CHOP was similar between male controls and those treated with 4-PBA (Fig. 5), whereas in females, ovariectomy significantly induced the expression of this apoptotic UPR marker, compared to female controls and controls receiving 4-PBA.

## Discussion

Accumulating evidence supports a role for ER stress in the pathogenesis of diabetes [12, 15, 16, 32]. High blood glucose levels stimulate a significant increase in insulin production by pancreatic beta cells, and the high demand may exceed the protein folding capacity of the beta cell ER, causing ER stress [15]. Cells respond to this stress by activating the UPR to restore the protein folding homeostasis. However, in conditions of chronic hyperglycemia, as observed in diabetes, homeostasis might not be restored and pancreatic beta cells may undergo apoptosis [33]. This creates a vicious cycle where surviving pancreatic beta cells attempt to compensate for the loss of beta cells by increasing their workload, further aggravating ER stress, leading to further beta cell apoptosis.

In this study we analyzed the expression of markers of the adaptive UPR (GRP78/GRP94, PDI) and of the apoptotic UPR (ATF4, CHOP) in the sexually dimorphic TH mice. Male TH mice became chronically hyperglycemic and female mice remained normoglycemic (Fig. 1, Fig. 2) [19, 20, 34, 35]. Normoglycemia in these mice is maintained without the need for elevated insulin levels, therefore pancreatic beta cell ER stress in females is not as prominent as in male mice. In agreement with this, we observed that the UPR is not significantly activated, as indicated by the quantification of the adaptive UPR markers GRP78/GRP94 and PDI (Fig. 4) (Supplemental material [29]).

Subsets of mice were administered the chemical chaperone 4-PBA, which alleviates ER stress by preventing misfolded protein aggregation [26]. GRP78 and GRP94 are central molecular chaperones in the UPR response which aid in proper protein folding [30]. In this study, GRP78/GRP94 markers were significantly increased in hyperglycemic male TH mice; however, treatment with 4-PBA significantly reduced their expression (Fig. 4). PDI is an enzyme that regulates protein folding by aiding in the formation of disulfide bonds. PDI plays a significant role in insulin maturation as the structure of insulin is dependent upon the presence of 2 disulfide bonds between the A and B chains, as well as an intra-chain disulfide bond in the A chain [36, 37]. Consistent with the expression of GRP78/GRP94, PDI was also significantly reduced in males treated with 4-PBA, compared to the untreated males (Supplemental material [29]). The 4-PBA treatment did not modulate the expression of the apoptotic UPR markers in pancreatic islets; however, it is interesting to note that ovariectomized females do show a significant increase in the expression of GADD153/CHOP, consistent with a reduction in beta cell mass (Figs. 3 and 5).

These results suggest that ER stress may play a role in the development of the chronic hyperglycemia observed in male TH mice (Fig. 1). These mice also show decreased glucose tolerance (Figs. 1 and 2) and have a significantly reduced beta cell mass (Fig. 3). Treatment with 4-PBA significantly reduces the expression of adaptive UPR markers but is not sufficient to improve beta cell health and function. This could result from the significant reduction of pancreatic beta cell mass in both male experimental groups, such that the surviving pancreatic beta cells are not capable of effectively regulating glucose homeostasis.

Women before menopause are protected from developing diabetes compared to men or to women after menopause [21-23]. Accumulating evidence suggests a protective role of estrogen in diabetes development [38, 39]. As women reach menopause, they experience a significant reduction in the levels of circulating estrogens, which is thought to contribute to the loss this protective effect [38-40]. Consistent with this, a prompt start of postmenopausal estrogen-replacement therapy has been shown to improve glucose tolerance [23, 41-45]. To determine whether estrogen depletion has an impact on glucose homeostasis in this rodent model, we ovariectomized female TH mice, as this procedure is a commonly used model of menopause/estrogen depletion [17, 46, 47]. While female sham-operated TH remain normoglycemic, we observed that ovariectomized females become chronically hyperglycemic. Additionally, we observed that ovariectomized female TH have a significant reduction in beta cell mass, insulin content, glucose tolerance, and plasma insulin levels, compared to female sham-operated mice (Figs. 1A, 2, and 3) (Supplemental material [29]). Our results support the hypothesis that a sharp decline of circulating estrogens induced by ovariectomy may play a role in developing chronic hyperglycemia in female TH mice in a pattern similar to what observed in TH males. The reduction in beta cell mass and consequent reduction in circulating insulin is likely a result of our experimental endpoint at 20 weeks of age for all mice (an advanced time point). This is similar to what observed in the later stages of type 2 diabetes [46, 47]. To our knowledge, this is the first study showing that reducing circulating estrogens in female TH mice impairs glucose homeostasis.

The results of this study are consistent with what we previously observed in a mouse model of hyperglycemia-induced atherosclerosis, the ApoE^−/−^:Ins2^+/Akita^ rodent model, where ovariectomy significantly changes the glycemic phenotype with ovariectomized female ApoE^−/−^:Ins2^+/Akita^ mice which remain chronically hyperglycemic and show signs of UPR activation [9, 10, 25]. It should be noted that the ApoE^−/−^:Ins2^+/Akita^ rodent model shows sexual dimorphism in glucose regulation similar to what is observed in TH mice [9, 10, 25].

Ovariectomy induced a significant increase in the molecular chaperones GRP78/GRP94, indicating that hyperglycemia is associated with an increase in ER stress. Consistent with what was observed in males, treatment with the ER stress alleviator 4-PBA reduced the expression of UPR markers but was not able to significantly improve and/or restore glucose homeostasis in the long term. This could be explained by the observed severe loss in beta cell mass that translates in the inability of the remaining pancreatic beta cells to effectively regulate glucose homeostasis [9].

We observed that 4-PBA transiently reduced fasting glucose levels in both male and female ovariectomized mice, although they remained hyperglycemic. This indicates that 4-PBA can improve glucose regulation but a more robust enhancement of the UPR may be required. Although this might be attained by increasing the dosage of 4-PBA, it is important to consider potential side effects and toxicity that this treatment might have. In fact, our metabolic parameters show that treatment with 4-PBA significantly increased liver weight. Furthermore, 4-PBA treatment may not enhance expression of other factors that may be required for an effective UPR, suggesting that other modulators such as estrogen might be needed. Previous analysis in the ApoE^−/−^:Ins2^+/Akita^ mice showed that chronically hyperglycemic male and female ovariectomized ApoE^−/−^:Ins2^+/Akita^ mice exhibited a significant increase in the expression of the apoptotic UPR marker GADD153/CHOP compared to female sham-operated ApoE^−/−^:Ins2^+/Akita^ mice who are normoglycemic [10, 25]. This is consistent with what we observed in ovariectomized TH females, where this pro-apoptotic marker was significantly increased. This may indicate that significant reductions of circulating estrogens may enhance the apoptotic response to ER stress and consequently affect glucose homeostasis.

While our results provide valuable insights, it is important to acknowledge the limitations of the mouse model. Like any animal model of disease, rodent models of diabetes recapitulate the main characteristics of the disease, but some features may appear exaggerated [38, 48, 49]. For example, fasting blood glucose levels are far higher in TH mice, compared to those in humans. Therefore, the range for normoglycemia and hyperglycemia in these mice is set at higher levels than that in humans. The TH animal model recapitulates various pathological features of type 2 diabetes, but only male mice develop chronic hyperglycemia and its related consequences. Female mice, even though they exhibit obesity and high insulin levels, remain normoglycemic [19, 35]. While we used this sexual dimorphism to evaluate sex as a biological variable in the development of diabetes, there are women who may develop diabetes before menopause onset (ie, women with polycystic ovary syndrome), and this model may not capture these potential risk factors [39]. A limitation of the ovariectomy model is that the drastic reduction in circulating estrogens does not mimic the potential effects of gradual hormonal changes observed during perimenopause [40].

Despite the limitations, experimental models of diabetes such as the TH mice, and the use of ovariectomized mice to investigate the effects of hormone changes, remain essential to explore the potential molecular mechanisms that underlie the development of this disease [38, 48, 49]. The observations in this study suggest that hyperglycemia triggers ER stress and the consequent UPR. While in this study the focus was on the potential role of ER stress in the development of hyperglycemia, we also observed that the presence or significant reduction of circulating estrogens may influence the UPR response. Future studies will explore the role of estrogen hormone therapies in the maintenance of glucose homeostasis and UPR activation. Taken together, this information will improve our understanding of the intricate interplay of sex hormones and the UPR in pancreatic beta cells, providing some insights on diabetes development and progression.

## Data Availability

Some or all datasets generated during and/or analyzed during the current study are not publicly available but are available from the corresponding author on reasonable request.

## Abbreviations

4-PBA: 4-phenylbutyric acid
ER: endoplasmic reticulum
GRP: glucose regulated protein
PDI: protein disulfide isomerase
TH: TALLYHO/JngJ
UPR: unfolded protein response
